# Retrospective Comparison of Nigeria’s Emergency Response Between the 2014 Ebola Outbreak and 2020 COVID-19 Pandemic

**DOI:** 10.7759/cureus.44300

**Published:** 2023-08-29

**Authors:** Ugochukwu A Eze, Adegboyega Alao, Oladipo V Akinmade, Dabota Y Buowari, Chinemerem D Onwuliri, Daniel C Gbujie

**Affiliations:** 1 Department of Ophthalmology, Federal Medical Centre, Asaba, Asaba, NGA; 2 School of Public Health, University of Suffolk, Ipswich, GBR; 3 Health Economics Division, Department of Economics, Kaduna State University, Kaduna, NGA; 4 Department of Medicine, Lagoon Hospital, Ikoyi, NGA; 5 United States Agency for International Development (USAID) Integrated Health Programs, Ministry of Health, Abakaliki, NGA; 6 Department of Accident and Emergency, University of Port Harcourt Teaching Hospital, Port Harcourt, NGA; 7 Department of Community Medicine, University of Nigeria Teaching Hospital, Enugu, NGA; 8 Department of Global Health, Southern New Hampshire University, Manchester, USA

**Keywords:** risk communication, selective pathogenicity, ebola outbreak, covid-19 pandemic, covid-19 retro

## Abstract

The 2014 Ebola Virus disease outbreak and the COVID-19 pandemic are prominent among the viral infectious diseases that threatened our existence in the last decade. We did a retrospective review of Nigeria’s responses during the two crises using different keywords: *pathogenicity, risk communication, data gathering, and vaccine issues*. These served as discussion points, and we ended by highlighting a few salient observations that should serve as reflection, learning points, and recommendations for better responses in the future. Based on these keywords, we noticed some differences in the two outbreaks, some of which affected the mode of response. At some point, Nigeria was commended for curtailing the Ebola outbreak. This was not the same with COVID-19 which is still very much with us. Also, the latter received more global attention. We then concluded the review by outlining salient points that should serve as reflection and learning points to serve as a guide for a better approach in future epidemics/disaster outbreaks.

## Introduction and background

There have been notable pandemics since the beginning of the twentieth century which include the 1918 "Spanish" flu caused by an influenza A (H1N1) virus, 1957 influenza A (H2N2) virus - Asian flu, 1968 influenza A (H3N2) virus - Hong Kong flu, 2002 severe acute respiratory syndrome (SARS) caused by SARS coronavirus, and the 2009 Swine flu caused by an influenza A (H1N1) virus [1]. More recently is the West African Ebola Virus Disease (EVD) which was an epidemic between 2013 and 2016 [1]. It was an outbreak with huge regional significance, claiming an estimated 11,300 lives in total [1]. More recently is the coronavirus disease (COVID-19) pandemic, caused by the severe acute respiratory syndrome coronavirus 2 (SARS-CoV-2) that emerged in Wuhan City, China in December 2019 and later spread to other parts of the world in 2020 [1,2]. The first case of COVID-19 in Nigeria was an Italian citizen who flew into the commercial city of Lagos from Milan, Italy while the first case of EVD in Nigeria was in a Liberian who also flew into Lagos [1,3]. The outbreak of Ebola started in Guinea in December 2013 and rapidly spread to Liberia and Sierra Leone in 2014 affecting at least eight countries, and culminated in the introduction of Ebola into Nigeria in July 2014 by an ill Liberian traveler to Lagos [3].

Both the EVD epidemic and the COVID-19 pandemic have been two major public health concerns in the last 10 years in Nigeria, and it will be beneficial to compare and learn from the emergency response approaches to the two diseases in Nigeria. Appraising the emergency responses to these two viral diseases can help improve the preparedness for future outbreaks.

Furthermore, it is on record that Nigeria was applauded locally and internationally for its response and containment of the Ebola disease outbreak [4,5]. The World Health Organization (WHO) on 20 October 2014 declared Nigeria Ebola having passed the stipulated minimum period of not having a new case after the last patient was discharged [4]. Nigeria was also commended by the WHO for its strong leadership and effective coordination of emergency responses such as the establishment of an Emergency Operations Centre whose activities helped contain the spread of the highly contagious disease (virus) [4,5]. On the other hand, the response to the COVID-19 outbreak in Nigeria was less prompt as the situation appeared more complex initially, perhaps because it was novel. Nigeria did not completely fail in its emergency responses, especially when we consider the case fatality rate (CFR) of the country, the apparent impact on the health system when compared with other countries with seemingly better health systems [6], and the increase in number or molecular laboratories in the country occasioned by the pandemic. It is important that we search for plausible reasons for this trend in order to emergency response in the future.

Nigeria is the most populous country in Africa (with a 2022 population estimate of over 206 million, the countries with the closes population are Ethiopia and Egypt each with about half the population of Nigeria) and a regional force on the Western Coast of Africa in terms of its fair contribution to the human resource for healthcare on the continent with about 26% of Africa's estimated 3.6 million health workers [7]. Therefore, it is pertinent that we share our experiences in order to guide the need for a regional emergency plan against outbreaks.

The WHO defines emergency preparedness as “the knowledge, capacities and organizational systems developed by governments, response and recovery organizations, communities and individuals to effectively anticipate, respond to, and recover from the impacts of likely, imminent, emerging, or current emergencies” [8]. This article aims to review, analyze, and compare the emergency preparedness and responses by Nigeria and the general population to the EVD epidemic and COVID-19 pandemic. It will also recommend strategies that could be useful in controlling the disease and possible future epidemics/pandemics. We shall approach this comparative retrospective review under the following subtopics: selective pathogenicity, communication, data gathering, and issues with vaccines.

## Review

Selective pathogenicity

While EVD and COVID-19 are both caused by highly infectious RNA viruses, EVD which belongs to the *Filoviridae *family is filamentous, non-segmented negative-stranded, causing hemorrhagic fevers and hemodynamic imbalance from fluid loss, hypotension, coagulopathy, and eventual shock [8]. COVID-19 is caused by an enveloped, single-stranded virus with a positive sense of the *Coronaviridae *family, whose infection could be asymptomatic, or cause mild, moderate to severe respiratory and even multisystem disease that may eventually result in multi-organ failure [9]. Consumption coagulopathy is also a common feature of both diseases. Though both viruses are highly infectious, they differ in CFR. The CFR for EVD is 50% while that for COVID-19 varies. However, as of 26 September 2022, the average global CFR for COVID-19 was 1.05% [6].

EVD was thought to be introduced to humans via contact with the body fluids of infected animals like fruit bats, apes, forest antelopes, and porcupines. However, human-to-human transmission is also possible through contact with blood, body fluids of a sick person, or contact with objects contaminated with body products (blood, feces, and vomitus) from a sick person or body of someone who has died from EVD [10]. Coronavirus is transmitted through airborne droplets to the nasal mucosa where the viruses replicate in the epithelial cells, causing inflammation and damage [10].

There are known pathogenic pathways through which viruses attack their host; fusion of the virus into the host cell (portal of entry of virion material), transmission and damage to the target tissues, virus increasing cellular replication and its dissemination to outlets for potential spread through shedding into the population environment [11]. However, some factors can influence the various pathogenic approaches stated above, they include proximity of pathogenic virus to target organs and tissue, cell susceptibility to viral replication, and virus susceptibility to the innate and adaptive immune system [11]. Like other viruses, once the coronavirus gains access to tissues it replicates locally using host genetic materials, spreads, and undergoes secondary replication. It has affinity to angiotensin-converting enzyme (ACE) receptors and mucosal surface but depending on host immunity, only about a third of infected individuals become symptomatic [9,11]. Also, due to immunological memory from previous exposure to a coronavirus, some circulating antibodies mount a sufficient immune response to the viral antigens [9,12]. Following the incubation period of 14 to 21 days, the Ebola virus infection progresses rapidly. Its symptoms are nonspecific and include fever, malaise, muscle pain, spread of cytokines, distorted clotting, severe bleeding tendencies, and other hematological abnormalities [8].

The targeted isolation and quarantine measures for EVD were sufficient to contain its spread within the country. These measures were applied from an advantaged position of having decades of experience with the disease, lessons learned from affected countries, and the preparedness of health authorities across different countries, Nigeria inclusive. On the contrary, COVID-19 came as a novel disease at the time, hence the initial uncertainty, limited information about the disease, and panic among stakeholders which led to the application of measures such as lockdowns, closure of borders, arbitrary fumigation of streets and buildings, and the application of different regimen for prevention and treatment based on assumptions and hunches by both professionals and the general public. To date there is no evidence to support the fumigation of traveler's luggage at Nigerian airports to prevent the spread of COVID-19 [13]; this was a compulsory practice for over a year, lasting even after the introduction of the vaccine. The COVID-19 pandemic led to the suspension of many routine healthcare services and elective procedures [14], resulting in increased morbidity and mortality from conditions that could have been easily managed from an outpatient clinic or prevented by applying routine primary health interventions. The selective pathogenicity for the two pathogens could have produced different results from the control measures instituted for both cases.

Communication and data gathering

Nigeria had time to prepare for Ebola while for COVID-19 the time was much shorter: considering that the first reports from China in December 2019 was barely two months before the first reported case in Nigeria. Although there was an interval of over five years between the spread of EVD from Liberia and COVID-19 from Italy to Nigeria, we attempt to share experiences around strategic communication and public communication as it relates to the Ebola virus disease and COVID-19 in Nigeria [15,16]. We had about 38 years of pre-warning for the Ebola virus disease which was first discovered in the dual outbreaks in South Sudan and the Democratic Republic of Congo, while the SARS-CoV-2 took only about 11 weeks from its discovery in Wuhan China before the first case in Nigeria was reported. Hence, there also lies an imbalance in time for disease-specific preparedness measures for both diseases.

Both outbreaks affected deep-seated cultural practices that encourage social gatherings in the country, by requiring the avoidance of physical contact to prevent further spread.

The International Health Regulations (2005) addressed 13 core capacities that should be available at every given point in time to ensure preparedness, efficient prevention, response, and control in the event of a public health emergency of international concern. Two of these core capacities are hinged on communication and information [17]. In the case of the EVD, the Liberian government did not provide information on the exposure of the index case who was traveling into Nigeria, while for COVID-19 the Chinese government delayed reporting the case. It also took significant time for the WHO to declare COVID-19 as a public health emergency of international concern. Therefore, the delayed response to both Ebola and COVID-19 left the public open to rumors.

Risk communication and community engagement are critical for a successful response to public health emergencies like the EVD epidemic and the COVID-19 pandemic [18,19]. Effective communication and engagement of stakeholders and community structure ensure that the right information about the pandemic is delivered to the public [18]. During both the EVD epidemic and the COVID-19 pandemic there were lots of rumors, myths, fake news, and wrong information about the disease which complicated the situation. For instance, during the EBV outbreak, there was a popular rumor that led to a large proportion of the general public bathing with and drinking salt water. This led to a high rate of hospitalizations and some reported cases of death across the country [20]. In a similar manner, during the COVID-19 pandemic, the cost of chloroquine quadrupled in some places with a significant number of people overdosing with chloroquine in a misguided bid to prevent infection. In response to this, regulatory agencies sent out warnings on the side effects and complications of chloroquine and hydroxychloroquine overdose [21]. Records show that standard surveillance and reporting systems were deployed following the report of the index case of EVD in Nigeria in July 2014, which contributed to its successful control [22]. This proactive approach in EVD response included the deployment and prompt training of personnel, risk communication via social media, organization of community and mass campaigns, interpersonal and house-to-house communication, and strategic meetings, among others [23]. On the other hand, the COVID-19 response had the media replete with misinformation and conspiracy theories within a very short period. Nigeria's media response was led by the Nigeria Center for Disease Control (NCDC) via social media, radio, and television stations.

Unlike that of EVD, where the professionals were allowed to take the lead in response and communication, the COVID-19 response in Nigeria witnessed the interference of politicians using their influence to make pronouncements which at times contradicted information from the field workers and the designated response agency. Different state governments had different response plans which were not necessarily evidence-based except for one state which had an epidemiologist as the deputy governor. In addition, due to international interests and significant donor funding, there was a lot of interference from politicians in the planning and implementation of response activities. There were a number of reported disputes between core professionals and their political chief executives particularly with respect to situation reports, budget allocation, and release for healthcare provision. There were also a lot of media accounts of the diversion of funds in the name of palliatives meant to cushion the effect of the COVID-19 pandemic [24,25].

The adoption of social media is fast growing with increasing access to mobile phones, the internet, data plans, and new applications; messages, memes, and posts can go viral in a matter of hours. This has been a blessing, as information can be transmitted to a wider audience in shorter periods of time, and users can be profiled based on location to tailor the type of information they receive. It is no doubt that the right information remained the most effective approach to curbing the EVD [26]. However, the challenge of misinformation on social media constituted the twin outbreak or pandemic as it were. For EVD, different myths and rumors constituted a barrier to its prevention and control across the world, some of which were picked from here-say but eventually made it to social media and became viral [27]. Same with COVID-19, a lot of myths and fake news were spread via social media, especially regarding the spread, treatment, and vaccines [28].

Issues with vaccines

It took 38 years since the first Ebola outbreak for an Ebola vaccine to be developed. One vaccine has been used in Ebola outbreaks while one is in clinical trial. This contrasts with the development of vaccines against COVID-19 which took about one year to develop and distribute [29].

While this advancement in response is majorly influenced by the level of technological advancement during the time of COVID-19, it is very important to highlight the level of global interest - the Ebola crisis has for a long time been a predominantly sub-Saharan epidemic with little impact on the global north, while COVID-19 was global with greater impact on the global north where most of the vaccine technology operates. This along with other economic and health equity concerns has affected availability, distribution, shelf-life, acceptability, and vaccination approaches in both cases. The impact of these factors in Nigeria may be minimal but not documented.

Neither the Ebovac^TM^ vaccine nor the J&J^TM^ Ebola vaccines have been used in Nigeria, hence we do not have empirical data on adverse events following vaccination. However, field reports from the Ebola belt have ranged from no effects to as mild as injection site pain, graduating to headaches and occasionally coma, which led to the administration of a half dose at some point. On the other hand, COVID-19 vaccines have been used in Nigeria with variable reported side effects which are milder than those recorded for EBV vaccines. The COVID-19 vaccine side effects range from weakness, headache, drowsiness, injection site pain, and lethargy which on some occasions lasted up to three days [30].

The cost per vial and availability of funding plays a significant role in vaccination and the approaches to vaccination. The Ebovac^TM^ vaccine is utilized only during outbreaks, using a ring vaccination and targeted approach - hence only cases, first and second contacts, and front-line/response workers are vaccinated. The second Ebola vaccine which is in trial is currently being tested for its potency creating immunity in the general population.

On the other hand, COVID-19 vaccines are much more available, with multiple funding streams and more variety. In Nigeria, vaccination (with Astra Zeneca® vaccine) started in March 2021, following the arrival on 2 March 2021 as a targeted approach that covered front-line workers, high-risk persons, and the elderly [31,32]. However, a mass vaccination approach has been employed, and this has metamorphosed into a fusion of approaches coined as the SCALES 3.0 strategy - Service delivery, Communication, Accountability, Logistics, Electronic management of immunization data, and Supportive supervision. COVID-19 vaccination has been integrated with routine immunization in principle, but like most interventions of its age, the finer details are still in process at the lower levels of the pyramid [33].

Ebola vaccination has not been implemented in Nigeria. However, COVID-19 vaccination commenced in Nigeria in March 2021 with demonstrable results such as drops in rates of infections, number of admitted cases, and mortalities [33]. According to WHO data, a total of 128,382,195 vaccine doses have been administered in Nigeria as of 6 August 2023 [34]. It was difficult to access data or information on the exact proportion of vaccination in Nigeria and its distribution across the regions of the country. However, a report from Nasarawa state in Nigeria in 2022 showed that about 70% of their eligible population had received the vaccination [35]. This figure is similar to the result of a population-based study in Nigeria where 66.2% of the respondents affirmed that they would receive an approved vaccine if advised by a health professional [36]. Furthermore, it has been reported in the literature that there is a disparity in access to vaccines between developed and underdeveloped countries. Despite the fact that WHO advocates 70% vaccination coverage across the globe, less than 1% of low-income countries and about 10% of lower-middle-income countries were currently vaccinated as of 2021 [37]. Nigeria was among the bottom 15 countries for COVID-19 vaccination [37].

Both have been met with some degree of hesitancy. We were unable to access peer-reviewed evidence on the current strategy and the vaccination monitoring platform for COVID-19 in Nigeria is not open source at the moment.

Lessons learned

This article highlighted some important differences in Nigeria’s responses to EVD and COVID-19 outbreaks. These observations include points discussed in subsequent paragraphs.

Effective leadership, coordination, and communication are crucial during emergency response. This was observed in the response to EVD, however the same was not sufficient in the COVID-19 pandemic response.

Professionals should be allowed to perform their statutory roles without undue political interference during disease outbreaks. This was the case with the EVD outbreak unlike the political interference experienced during the COVID-19 pandemic in Nigeria.

The role of focused community mobilization of critical stakeholders and opinion leaders is key in any health advocacy. This can be done through improved communication channels like the use of local radio and television stations that can reach remote places.

No two public health emergencies are the same. While professionals operate using certain outlined evidence-based principles, there is no "one cap fits all" intervention. Beyond the application of general principles, disaster managers should respond to events while maintaining a high level of preparedness for any contingency.

The interest of the global community in response to the COVID-19 pandemic is quite different from the experience during the EVD outbreak. The reasons for this may be beyond the scope of this retrospective review. However, it is important to emphasize the pivotal role that health equity, socioeconomic determinants of health, and political economy play in response to global public health threats.

With technological advancement, the world has witnessed an unprecedented feat in vaccine production and trial in the COVID-19 pandemic. Unfortunately, there is a wide gap in access to vaccines and resource allocation across nations. First, the production and cost of these vaccines are unevenly distributed, with many countries holding on to vaccines and eventually donating them at a time when they were near expiry. The synergy between health systems can help to bridge this gap. In addition, developing nations like Nigeria would greatly benefit from venturing into local vaccine production.

Lastly, national response should focus on local solutions to global problems when adopting external approaches, their implementation should be context-specific.

## Conclusions

This article reviewed, analyzed, and compared the emergency preparedness and responses by Nigeria and the general population to the EVD epidemic and COVID-19 pandemic. As expected, we noticed the marked differences in the natural biology of the two viruses, hence their selective pathogenicity. There were some similarities and differences in the communication of these outbreaks to the general population while a lot of political interference was experienced by the frontline health workers during the ongoing COVID-19 pandemic. Salient points have been outlined which include closing communication gaps, professionalism, and encouraging evidence-based and innovative local solutions to outbreaks (investment into vaccine production in Nigeria), which would come in handy for future public health emergency response.

## References

[1] Abayomi A, Balogun MR, Bankole M (2021). From Ebola to COVID-19: emergency preparedness and response plans and actions in Lagos, Nigeria. Global Health.

[2] Anyanwu MU, Festus IJ, Nwobi OC (2020). A perspective on Nigeria’s preparedness, response and challenges to mitigating the spread of COVID-19. Challenges.

[3] Musa E, Nasidi A, Shuaib F (2016). Nigeria’s Ebola outbreak response: lessons for future epidemic preparedness. Arch Med.

[4] (2022). World Health Organization Declares Nigeria Ebola-Free. https://www.afro.who.int/news/who-declares-nigeria-ebola-free.

[5] (2022). How Nigeria won the fight against Ebola. https://reliefweb.int/report/nigeria/iddr2016-how-nigeria-won-fight-against-ebola.

[6] (2022). COVID-19 Visualizer. https://www.covidvisualizer.com/.

[7] (2022). WHO. Framework for a public health emergency operations centre. Geneva: World Health Organization. https://www.who.int/publications/i/item/framework-for-a-public-health-emergency-operations-centre.

[8] Sullivan N, Yang ZY, Nabel GJ (2003). Ebola virus pathogenesis: implications for vaccines and therapies. J Virol.

[9] Tyrrell DAJ, Myint SH (1996). Coronaviruses. Medical Microbiology. 4th edition.

[10] (2022). Ebola Virus Disease. WHO.

[11] Baron S, Fons M, Albrecht T (1996). Viral Pathogenesis. Medical Microbiology. 4th edition.

[12] Yaqinuddin A (2020). Cross-immunity between respiratory coronaviruses may limit COVID-19 fatalities. Med Hypotheses.

[13] (2023). Safety tunnels fumigation useless in fight against COVID-19 - experts. https://www.vanguardngr.com/2020/08/safety-tunnels-fumigation-useless-in-fight-against-covid-19-experts/.

[14] Ally N, Ismail S, Naidu N (2023). Impact of COVID-19 on ophthalmic surgical procedures in sub-Saharan Africa: a multicentre study [preprints]. Lancet.

[15] Althaus CL, Low N, Musa EO, Shuaib F, Gsteiger S (2015). Ebola virus disease outbreak in Nigeria: transmission dynamics and rapid control. Epidemics.

[16] Amzat J, Aminu K, Kolo VI, Akinyele AA, Ogundairo JA, Danjibo MC (2020). Coronavirus outbreak in Nigeria: burden and socio-medical response during the first 100 days. Int J Infect Dis.

[17] (2022). WHO International Health Regulations. Third Edition. https://www.who.int/publications/i/item/9789241580496.

[18] Kakaire CN, Reader S (2022). Coordinating risk communication and community engagement for a better COVID-19 response in Eastern and Southern Africa. Pan Afr Med J.

[19] Gonah L (2020). Key considerations for successful risk communication and community engagement programmes during the COVID-19 pandemic and other public health emergencies. Ann Glob Health.

[20] (2023). Ebola sparks panic across Nigeria as citizens scramble for salt-water bath “remedy”. https://www.premiumtimesng.com/news/166257-ebola-sparks-panic-across-nigeria-as-citizens-scramble-for-salt-water-bath-remedy.html?tztc=1.

[21] (2023). FDA cautions against use of hydroxychloroquine or chloroquine for COVID-19 outside of the hospital setting or a clinical trial due to risk of heart rhythm problems. https://www.fda.gov/drugs/drug-safety-and-availability/fda-cautions-against-use-hydroxychloroquine-or-chloroquine-covid-19-outside-hospital-setting-or.

[22] Gorman S (2013). How do we perceive risk?: Paul Slovic’s landmark analysis. https://scienceblogs.com/thepumphandle/2013/01/16/how-do-we-perceive-risk-paul-slovics-landmark-analysis-2.

[23] Maduka O, Maleghemi S, Komakech W (2016). Effective risk communication and contact tracing for Ebola virus disease prevention and control - Experiences from Port Harcourt, Nigeria. Public Health.

[24] (2023). PDP Demands Probe into FG’s COVID-19 Intervention Fund. https://www.premiumtimesng.com/news/headlines/456700-how-covid-19-palliatives-were-hijacked-distributed-among-party-loyalists-report.html?tztc=1.

[25] (2023). COVID-19 lockdown: How ‘diversion’ denied many poor, vulnerable persons govt’s food. https://punchng.com/covid-19-lockdown-how-diversion-denied-many-poor-vulnerable-persons-govts-food/.

[26] Seytre B (2016). The wanderings of the communication on the Ebola virus disease [Article in French]. Bull Soc Pathol Exot.

[27] Kasereka MC, Hawkes MT (2019). 'The cat that kills people:' community beliefs about Ebola origins and implications for disease control in Eastern Democratic Republic of the Congo. Pathog Glob Health.

[28] Eze UA, Ndoh KI, Kanmodi K (2021). COVID-19 crisis in Africa: revisiting the contributing factors. Annals of Public Health Issues.

[29] Ebola ça Suffit Ring Vaccination Trial Consortium (2015 (2022). COVID-19 tracker, supported by the McGill University Interdisciplinary Initiative in Infection and Immunity (MI4). https://covid19.trackvaccines.org/country/nigeria/.

[30] (2022). National Primary Health Care Development Agency NPHCDA. BMJ.

[31] (2023). Nigerian health workers take country’s first COVID-19 vaccine. https://www.afro.who.int/news/nigerian-health-workers-take-countrys-first-covid-19-vaccine.

[32] (2023). COVID-19 vaccines shipped by COVAX arrive in Nigeria. https://www.unicef.org/wca/press-releases/covid-19-vaccines-shipped-covax-arrive-nigeria.

[33] (2022). Nigeria Centers for Disease Control NCDC COVID-19 Nigeria Microsite. https://covid19.ncdc.gov.ng/.

[34] (2023). Nigeria: WHO Coronavirus Disease Dashboard. https://covid19.who.int/region/afro/country/ng.

[35] (2023). Making Good on a Promise: How Nasarawa State Vaccinated Seventy Percent of its Eligible Population. https://articles.nigeriahealthwatch.com/making-good-on-a-promise-how-nasarawa-state-vaccinated-seventy-percent-of-its-eligible-population/.

[36] Eze UA, Ndoh KI, Ibisola BA (2021). Determinants for acceptance of COVID-19 vaccine in Nigeria. Cureus.

[37] Yarlagadda H, Patel MA, Gupta V, Bansal T, Upadhyay S, Shaheen N, Jain R (2022). COVID-19 vaccine challenges in developing and developed countries. Cureus.

